# Supplementary material for: Reverse translation of adverse event reports paves the way for de-risking preclinical off-targets
Source: eLife. 2017 Aug 8;6:e25818. doi: 10.7554/eLife.25818 (PMC5548487; doi:10.7554/eLife.25818)
Supplement: Supplementary file 2. — ‘Physician’ was used as the reference level in Models 2 and 4 for the variables involving the occupation of the reporting party. Significance codes: 0 ‘***’ 0.001 ‘**’ 0.01 ‘*’ 0.05 ‘.’ 0.1 ‘‘1. The ‘:’ symbol separating two variable names denotes a variable representing an interaction between these two variables. DOI: http://dx.doi.org/10.7554/eLife.25818.015 [file elife-25818-supp2.docx]

| **Variable** | **Coefficient** | **Std. Error** | **z-value** | **Pr(>\|z\|)** |
| --- | --- | --- | --- | --- |
| **Model 1** | | | | |
| (Intercept) | 3.863249 | 0.002389 | 1617.3 | < 2e-16 *** |
| has_celecoxib | -1.923659 | 0.013752 | -139.9 | < 2e-16 *** |
| **Model 2** | | | | |
| (Intercept) | 3.918092 | 0.005084 | 770.728 | < 2e-16 *** |
| has_celecoxib | -0.631029 | 0.070136 | -8.997 | < 2e-16 *** |
| has_celecoxib:occupationConsumer | 0.091792 | 0.085331 | 1.076 | 0.2821 |
| has_celecoxib:occupationLawyer | -2.968832 | 0.074822 | -39.679 | < 2e-16 *** |
| has_celecoxib:occupationOtherHealthP  rofessional | 0.211581 | 0.130436 | 1.622 | 0.1048 |
| has_celecoxib:occupationPharmacist | 0.655089 | 0.254653 | 2.572 | 0.0101 * |
| **Model 3** | | | | |
| (Intercept) | 3.831648 | 0.002582 | 1484.22 | < 2e-16 *** |
| has_celecoxib | -2.308923 | 0.014511 | -159.12 | < 2e-16 *** |
| has_celecoxib:before_2005 | 1.570875 | 0.086980 | 18.06 | < 2e-16 *** |
| **Model 4** | | | | |
| (Intercept) | 3.923403 | 0.005394 | 727.338 | < 2e-16 *** |
| has_celecoxib | -0.631191 | 0.077394 | -8.156 | 3.48e-16 *** |
| has_celecoxib:before_2005 | -0.034115 | 0.182452 | -0.187 | 0.85167 |
| has_celecoxib:occupationConsumer | 0.168359 | 0.093533 | 1.800 | 0.07186 . |
| has_celecoxib:occupationLawyer | -2.981812 | 0.081662 | -36.514 | < 2e-16 *** |
| has_celecoxib:occupationOtherHealthP  rofessional | 0.158893 | 0.143150 | 1.110 | 0.26701 |
| has_celecoxib:occupationPharmacist | 0.712087 | 0.301353 | 2.363 | 0.01813 * |
| has_celecoxib:before_2005:occupation  Consumer | -0.747393 | 0.230301 | -3.245 | 0.00117 ** |
| has_celecoxib:before_2005:occupation  Lawyer | 2.397864 | 0.753976 | 3.180 | 0.00147 ** |
| has_celecoxib:before_2005:occupation  OtherHealthProfessional | 0.280779 | 0.34788 | 0.807 | 0.41960 |
| has_celecoxib:before_2005:occupation  Pharmacist | -0.218329 | 0.568187 | -0.384 | 0.70079 |
