# Supplementary material for: Reverse translation of adverse event reports paves the way for de-risking preclinical off-targets
Source: eLife. 2017 Aug 8;6:e25818. doi: 10.7554/eLife.25818 (PMC5548487; doi:10.7554/eLife.25818)
Supplement: Supplementary file 3. — The Oracle algorithms for detection of duplicate reports and determination of generic drug names yield slightly different counts than does the data preparation method described in the present paper, although computed adverse event rates are virtually identical. The columns for N, E, and EB05 shown in the table are as calculated by the Oracle program. Columns represent: Drug (Generic name) PT (MedDRA Preferred Term) N (Number of reports including both Drug and PT, 1997–2015, in the Oracle Health Sciences curation of FAERS) E (Expected value of N if Drug and PT are independent within each stratum, where strata are defined by all combinations of gender, age group and year of report) RR (Relative Reporting Rate = N/E) EBGM (Empirical Bayes Geometric Mean of estimated disproportionality) EB05 (Lower limit of Bayesian 90% confidence interval of true disproportionality) EB95 (Upper limit of Bayesian 90% confidence interval of true disproportionality) DOI: http://dx.doi.org/10.7554/eLife.25818.016 [file elife-25818-supp3.docx]

| **Generic_Name** | **PT** | **N** | **E** | **RR** | **EBGM** | **EB05** | **EB95** |
| --- | --- | --- | --- | --- | --- | --- | --- |
| Axitinib | Hypertension | 238 | 46.204 | 5.151 | 5.05 | 4.535 | 5.611 |
| Pazopanib | Hypertension | 572 | 121.472 | 4.709 | 4.674 | 4.361 | 5.004 |
| Sorafenib | Hypertension | 775 | 176.297 | 4.396 | 4.373 | 4.121 | 4.638 |
| Regorafenib | Hypertension | 149 | 33.467 | 4.452 | 4.334 | 3.781 | 4.949 |
| Ponatinib | Hypertension | 110 | 23.694 | 4.643 | 4.47 | 3.813 | 5.214 |
| Cabozantinib | Hypertension | 61 | 14.38 | 4.242 | 3.991 | 3.22 | 4.9 |
| Vandetanib | Hypertension | 25 | 9.597 | 2.605 | 2.394 | 1.709 | 3.28 |
| Sunitinib | Hypertension | 969 | 295.329 | 3.281 | 3.271 | 3.102 | 3.448 |
| Nintedanib | Hypertension | 17 | 12.238 | 1.389 | 1.312 | 0.871 | 1.914 |
| Ibrutinib | Hypertension | 57 | 58.753 | 0.97 | 0.96 | 0.769 | 1.187 |
| Tofacitinib | Hypertension | 77 | 101.853 | 0.756 | 0.752 | 0.622 | 0.903 |
| Ruxolitinib | Hypertension | 49 | 86.698 | 0.565 | 0.563 | 0.443 | 0.707 |
| Erlotinib | Hypertension | 183 | 327.814 | 0.558 | 0.558 | 0.493 | 0.629 |
| Dasatinib | Hypertension | 64 | 111.323 | 0.575 | 0.573 | 0.465 | 0.7 |
| Bosutinib | Hypertension | 5 | 13.858 | 0.361 | 0.362 | 0.17 | 0.695 |
| Gefitinib | Hypertension | 22 | 78.722 | 0.279 | 0.281 | 0.196 | 0.392 |
| Afatinib | Hypertension | 5 | 18.26 | 0.274 | 0.279 | 0.131 | 0.536 |
| Crizotinib | Hypertension | 9 | 50.167 | 0.179 | 0.183 | 0.104 | 0.304 |
| Ceritinib | Hypertension | 2 | 6.707 | 0.298 | 0.309 | 0.097 | 0.79 |
